# Chemometric Analysis of Lavender Essential Oils Using Targeted and Untargeted GC-MS Acquired Data for the Rapid Identification and Characterization of Oil Quality

**DOI:** 10.3390/molecules22081339

**Published:** 2017-08-11

**Authors:** David J. Beale, Paul D. Morrison, Avinash V. Karpe, Michael S. Dunn

**Affiliations:** 1 Land and Water, CSIRO, P.O. Box 2583, Dutton Park, QLD 4102, Australia; 2 Australian Centre for Research on Separation Science, School of Applied Sciences, RMIT University, Melbourne, VIC 3001, Australia; paul.morrison@rmit.edu.au; 3 Faculty of Science, Engineering and Technology, Swinburne University of Technology, Hawthorn, VIC 3122, Australia; akarpe@swin.edu.au; 4 Technical Development, Seqirus, 63 Poplar Road, Parkville, VIC 3052, Australia; michael.dunn@seqirus.com.au

**Keywords:** environmental adulteration, geographical adulteration gas chromatography, mass spectrometry, predictive chemometric modelling

## Abstract

Standard raw material test methods such as the ISO Standard 11024 are focused on the identification of lavender oil and not the actual class/quality of the oil. However, the quality of the oil has a significant effect on its price at market. As such, there is a need for raw material tests to identify not only the type of oil but its quality. This paper describes two approaches to rapidly identifying and classifying lavender oil. First, the ISO Standard 11024 test method was evaluated in order to determine its suitability to assess lavender oil quality but due to its targeted and simplistic approach, it has the potential to miss classify oil quality. Second, utilizing the data generated by the ISO Standard 11024 test methodology, an untargeted chemometric predicative model was developed in order to rapidly assess and characterize lavender oils (*Lavandula angustifolia* L.) for geographical/environmental adulteration that impact quality. Of the 170 compounds identified as per the ISO Standard 11024 test method utilizing GC-MS analyses, 15 unique compounds that greatly differentiate between the two classes of lavender were identified. Using these 15 compounds, a predicative multivariate chemometric model was developed that enabled lavender oil samples to be reliably differentiated based on quality. A misclassification analysis was performed and it was found that the predictions were sound (100% matching rate). Such an approach will enable producers, distributers, suppliers and manufactures to rapidly screen lavender essential oil. The authors concede that the validation and implementation of such an approach is more difficult than a conventional chromatographic assay. However, the rapid, reliable and less problematic screening is vastly superior and easily justifies any early implementation validation difficulties and costs.

## 1. Introduction

Adulteration of a pure substance occurs when it is intentionally altered by the addition of foreign materials or exposed to an environment that would induce a change [1]. In the context of the essential oil industry, adulteration can be used as a means to increase product yields, decrease production costs and/or enhance the perceived quality of the final product, with increased profit being the principal incentive [2,3]. Underlying this is the low manufacturing yields in comparison to the costs of high quality oil production and the sourcing of raw materials. There is also a lack of regulation regarding the classification and labelling of essential oils, which is highlighted by the difficult challenge of identifying adulterated finished products. Compounding the issue even further, there is some inherent natural variation within oils as a result of the environmental and geological conditions during plant growth and harvesting [4,5,6,7,8,9].

For the essential oil of lavender, adulteration has been widely observed. The most common form of adulteration is where oil from the English lavender plant (*L. angustifolia* Miller) is adulterated with the essential oil from the much cheaper sterile hybrid, lavandin (*Lavandula* × *Intermedia*) [10]. In comparison to lavender, lavandin essential oil does have a similar scent but contains a greater level of terpenes (mostly camphor) that gives the oil a sharper overtone. The difference between the two oils is easily detectable by scent, however, when blended together, the sharper tones of lavandin are diluted, thus, making it difficult to detect by scent alone. At approximately $38 AUD/kg for lavandin essential oil compared to $251 AUD/kg for lavender essential oil, the incentive to adulterate oil is high considering the lack of regulation and the low probability of the adulterant being identified [1]. Another form of adulteration amongst essential oils is referred to as environmental adulteration, where oils from a lesser-quality yielding geographical location is labelled as being derived from a higher-quality/valued geographical location. For example, essential oils harvested from some European countries are typically sold at a premium compared to oils harvested from Asian nations, not only because of reduced labor costs in Asia but because of the increased oil quality due to environmental and geographical factors [9]. Of note, Hassiotis et al. [11] concluded that within a geographical location, essential oil quality can also varying due to habitat and diurnal changes at the site of production. The time of day plants are harvested [12], the crop duration and number of multiple harvests [13], and postharvest storage conditions [14] can also result in reduced oil quality. However, skilled blenders of oils are capable of making blends that are almost indistinguishable from the pure oil variety using conventional laboratory testing techniques such as Gas Chromatography (GC). With more sophisticated testing, it is possible to distinguish the pure from the adulterated oils and also identify ‘*multiple geographically sourced*’ blended oils but this requires capabilities not often available to most testing laboratories [15].

Modern methods routinely used for determining the composition and quality of essential oils include GC, high performance liquid chromatography (HPLC), Mass Spectrometry (MS) and Nuclear Magnetic Resonance (NMR) spectroscopy [2]. Chromatographic techniques such as GC and HPLC are used to separate essential oils into their individual constituents so that they can be identified and potentially quantified by a coupled detector such as a MS. The GC technique lends itself to the analysis of essential oils, as it is ideal for the analysis of volatile organic compounds. When used in conjunction with MS and NMR spectroscopy, GC has revolutionized the detection of minor chemical constituents within essential oils. MS looks at the fragmentation patterns of compounds under ionizing conditions, and this information is used to deduce their structures. NMR elucidates the structures of molecules by examining the environment of specific atoms such as ^1^hydrogen and ^13^carbon within a molecule. The sensitivity of analytical techniques for organic compounds has increased dramatically over recent years to the point where even trace constituents, including pollutants such as pesticides, can be detected [2]. Furthermore, the development of chiral GC-MS techniques has been found to be a useful approach for the authentication of essential oils [1,16]. The use of a chiral column in GC enables the analyst to separate enantiomers from one another and determine their unique ratios. The ratio of enantiomers within an essential oil is indicative of its biological origin and thus can provide strong evidence of any adulteration. Chiral GC-MS has been shown to detect lavender oil adulterated with synthetic linalool and linalyl acetate [17,18,19], lavandin oil [16,20] and grapefruit oil [21]. Analysis of the minor organic components within the oil can also be used to distinguish between geographical locations or harvesting season [9].

ISO standard 11024 [22,23] details the GC protocol for obtaining chromatographic profiles of essential oils, detailing the compounds and representative characteristics that can be used to assess oil quality. This requires an authentic reference standard to which unknown oils are assessed against, after chromatographic integration and peak alignment. The approach outlined in the standard requires the use of a skilled analytical chemist, and the integration and comparison between samples can be a time consuming process if multiple samples from multiple batches are to be analyzed. One approach to expedite the screening of oils and make them available for sale faster is through the use of chemometric data analysis techniques. Chemometrics has been applied to MS acquired essential oil data to monitor mixtures of oils in foods, cosmetics or pharmaceuticals, assess quality and authentication [24]. The application of chemometrics in this context relies on using chromatographic features to develop predicative class models that can rapidly assess GC-MS derived data and categories samples based on quality.

As such, the study herein describes the utilization of a simple GC-MS method followed by a chemometric analysis for the identification and characterization of two different grades of lavender oil. The two oil variants, referred to as ‘*Essential oil’* and ‘*Garden Oil’*, were sourced from an essential oil distributor who is seeking a fast screening method that is able to reliability qualify oils as either high quality or low grade. The current approach used by the distributor is to analyze new sample batches for the seven target compounds of interest as identified in the ISO standard, namely: 1,8-cineole, *cis*-β-ocimene, linalool, 1-octen-3-yl acetate, camphor, linalyl acetate and lavandulyl acetate. These target compounds are common features in lavender essential oils [1]. While this approach serves its purpose, there have been incidences of miss-graded oils where higher quality oils have been graded as low grade. Such misclassifications can have a negative impact on financial metrics. Therefore, GC-MS derived data from a targeted and untargeted analysis were used to develop two predicative models. The models were assessed for their suitability to predict and classify new samples, validated using 9 additional unknown samples which were subsequently characterized.

## 2. Results and Discussion

An Australian lavender oil distributor provided a reference essential oil standard (natural *L. angustifolia* L. oil, CAS Number 8000-28-0) for analysis. This reference standard is used by the distributor to characterize oils received from France, Bulgaria, Indonesia and Cambodia. It is important to note that all the oils used for the experiments detailed herein were derived from *L. angustifolia*. In addition, the variation of oil composition has been reported to be within industry acceptable limits for the identification of lavender essential oil. Due to this, the distributer supplies the oils originating from France and Bulgaria as either certified essential oils and are distributed as high quality oil, having greater olfactory notes. The oils that originate from Indonesia and Cambodia are of a ‘*perceived lower quality*’. Both claim to be derived from the high quality yielding lavender species of *L. angustifolia* but there is a distinct difference in the quality of the scents produced. The exact cause for the lower quality oil is not known, though it is most likely due to its geographical region and/or adulteration. All that is known is that from the perspective of the distributor, the lower grade lavender oil is considered as an ‘*adulterated oil type*’ due to its perceived scent quality. Whatever the cause for the drop in quality be it geographical, adulteration or a combination of both, by industry standards they are considered adulterated and will be used as a positive control for such throughout this study.

### 2.1. Targeted Analysis

The ISO Standard 11024 is a targeted analysis that focuses on the percentage abundance of seven target components within a sample of lavender essential oil sample. The target components are 1, 8-cineole, *cis*-β-ocimene, linalool, 1-octen-3-yl acetate, camphor, linalyl acetate and lavandulyl acetate. This section investigates the limits of applying such a targeted simplistic approach to a highly complex biological sample that is subject to high levels of natural variation.

#### 2.1.1. Lavender Reference Standard Assessment Using the ISO Standard 11024

The lavender reference standard was analyzed for the presence of 1,8-cineole, *cis*-β-ocimene, linalool, 1-octen-3-yl acetate, camphor, linalyl acetate and lavandulyl acetate. Analytical conditions were optimized in order to acquire reproducible and valid data for the analysis and subsequent predictive modelling. Alkane internal standards (C_8_–C_22_) were used during the analyses for the determination of linear retention indices (LRI) and assay performance. Relative standard deviations (%RSDs) were calculated to be between 3.6–13.3% for the 13 individual alkane standards, with a mean %RSD of 5.7 ± 2.5 (*n* = 7). The target compounds of 1,8-cineole, *cis*-β-ocimene, linalool, 1-octen-3-yl acetate, camphor, linalyl acetate and lavandulyl acetate were found to account for ca. 74.5% (1.8% RSD) of each oil analyzed in terms of peak area. Furthermore, Table 1 illustrates that the relative percentage peak area of each target component meet the requirements outlined in the ISO standard 11024 [22,23]. Table 1 also details the retention time and calculated LRI of the target compounds.

#### 2.1.2. Comparison of Lavender Oil Samples Using the Targeted ISO Standard 11024 Test Method

**Figure 1:**
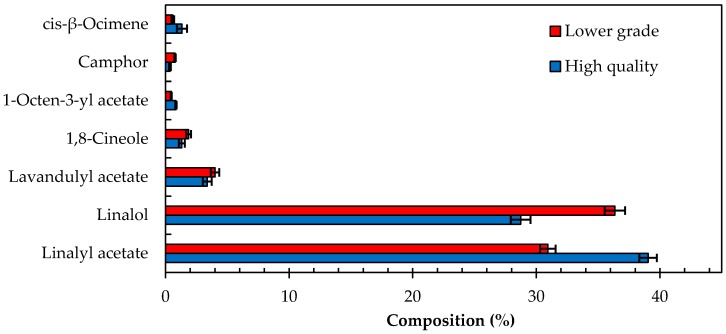
Comparison of target compounds found in the reference sample in the high quality and lower grade lavender oil samples analyzed. The data presented is derived from the analysis of 54 oil samples, 30 qualified as high quality and 24 qualified as lower grade lavender oil. Error bars are indicative the standard deviation of the cohort.

Analysis of 30 high quality and 27 lower grade oils as per ISO Standard 11024 indicated that the seven target compounds used to characterize the lavender oil samples were all present (Figure 1). Statistical analysis of these compounds and their relative composition in the high quality and lower grade oils concluded that these oils are significantly different (Table 2), albeit within acceptable compositional ranges as per the ISO Standard 11024. The ratio of *t*-statistic and t-critical values (*t*-stat: *t*-crit.) in two sample *t*-Test analysis and the ratio of *F*-value and *F*-critical value (*F*: *F*-crit.) in a single factor ANOVA analysis indicate that the mean values of the target compounds within the two varieties of lavender oil were significantly different from each other thus indicating the varying quality in the two oil cohorts (Table 2). However, as described in the following section, the utilization and application of the target compounds alone in a predicative model was found to be unreliable.

As illustrated in Table 2, the %RSD of the major compounds, linalyl acetate and linalol, were 4 and 4–6% for higher and lower graded oils, respectively. The %RSD for other compounds, which are reportedly found at lower concentrations varied significantly with a range of 14–64%. However, it is noteworthy to mention that both oil classes were within acceptable percentage composition ranges that are classified as lavender essential oils.

Using ISO Standard 11024, all lavender samples tested were successfully identified as being lavender essential oil. However, the ISO Standard 11024 cannot reliably distinguish between the two classes which as pointed out earlier in the introduction section of this report, which is of great monetary importance to the industry. The specification limits applied in the ISO Standard 11024 for the percentage abundance of the targeted compounds are not stringent enough to differentiate between the two classes. A simple solution would be to recommend that tighter specifications be set for the targeted compounds in the ISO Standard 11024 test method but this would be a great over simplification. Applying such stringent specifications to only a small number of target compounds within such a variable and complex matrix will be problematic for the industry to administer as the probability for miss identifications will be significant and costly.

### 2.2. Assessment of the Targeted ISO Standard 11024 Test Method Using a Predictive Model

The data acquired from the targeted ISO Standard 11024 GC-MS analysis of the essential oil samples was further analyzed using multivariate discrimination techniques, such as principle component analysis (PCA) and partial least squares-discrimination analysis (PLS-DA). This expands on the simplistic approach of individual percentage abundance specifications for each targeted compound and focuses on correlations between the percentage abundance of the targeted compounds.

**Figure 2:**
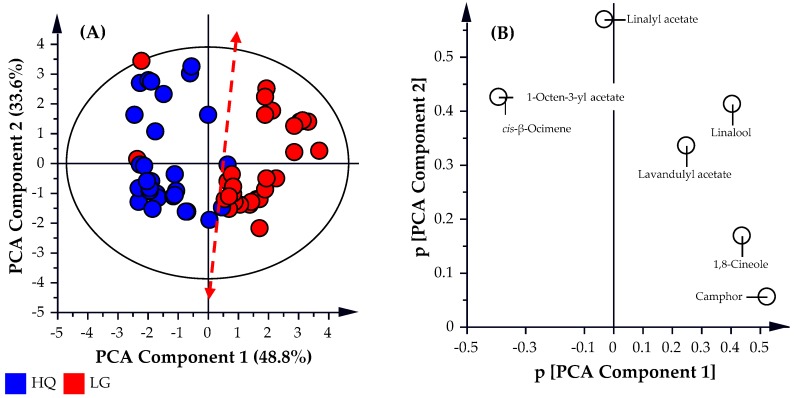
(**A**) PCA Scatter plot and (**B**) PCA Loadings Scatter Plot of the analyzed samples by targeted GC-MS.

As illustrated in Figure 2, all the high-quality oil samples analyzed (represented by the blue circles) were predominately distributed in the left hemisphere of the PCA scatter plot (Figure 2A). Conversely, the lower grade oil samples (represented by the red circles) were present predominately in the right hemisphere of the PCA scatter plot, with a few samples positioned in the left hemisphere. This indicates reasonable separation between the two sample cohorts. The *R*^2^*X*, and *Q*^2^ values obtained for the PCA analysis were observed to be 0.825 and 0.580, respectively, thereby providing a weaker predictive model. This is representative of a model that fits the data well but has weak-to-fair predictive capabilities (*Q*^2^ ~ 0.6). As illustrated by the *t*-stat.: *t*-crit. values in the two sample *t*-test analysis and the *F*:*F*-crit. values in the single factor ANOVA analysis values in Table 2, the variation between samples within each cohort is significant and statistically the two cohorts are not classed as the same. The PCA analysis could not distinguish between the two classes with reliable predictability possibly due to the limited focus on the seven targeted compounds. This shows that the simple application of more stringent specifications to the percentage abundance of the seven targeted compounds to distinguish between oil classes will be problematic. To develop a predictive model with a stronger predictive capability (*Q*^2^), the analysis needs to be broadened beyond the seven targeted compounds and include all identified compounds (170) found in the GCMS analysis of the lavender essential oil samples.

### 2.3. Development of a Non-Targeted Predictive Model

**Figure 3:**
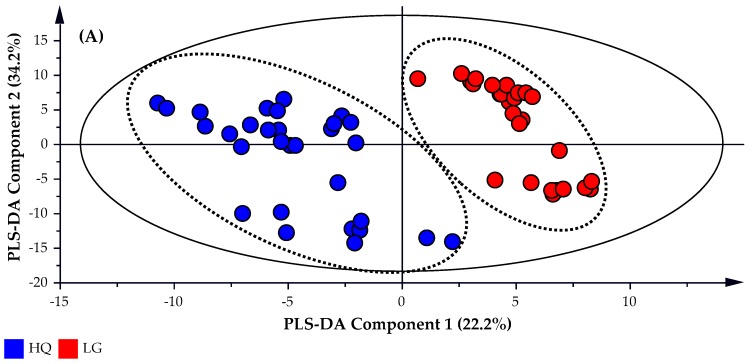

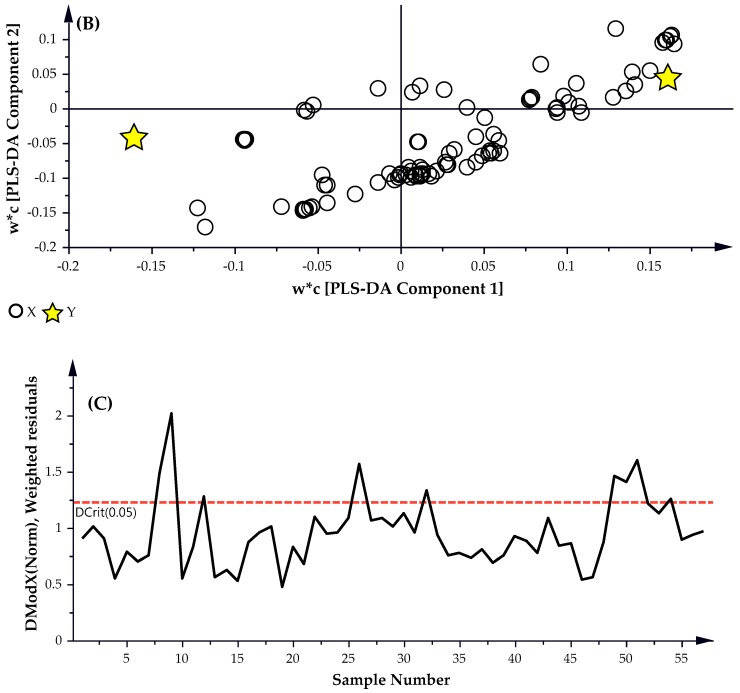
(**A**) PLS-DA Scatter plot, (**B**) PLS-DA Loadings Scatter Plot and (**C**) DModX’ or ‘Distance of observation’ plot of the analyzed lavender oil samples by untargeted GC-MS.

A partial least square discriminate analysis (PLS-DA) is applied to the untargeted GC-MS data set which identified 170 possible compounds. The subsequent PLS-DA Score and Scatter plots were generated using the untargeted data and are presented in Figure 3A,B, respectively. A Distance of Observation (DModX) analysis was used to identify and eliminate any outliers that may be present. DModX is the normalized observational distance between a variable set and X modal plane and is proportional to the variable residual standard deviation. ‘DCrit (critical value of DModX)’, derived from the F-distribution calculates the size of the observational area under analysis. As illustrated in Figure 3C, the DModX plot of the PLS-DA data indicated that no sample exceeded the threshold for rejecting a sample. Where the threshold for a moderate outlier to be rejected is identified when the sample DModX value is twice the DCrit at 0.05, which in this instance was 2.452 (DCrit = 1.226).

**Figure 4:**
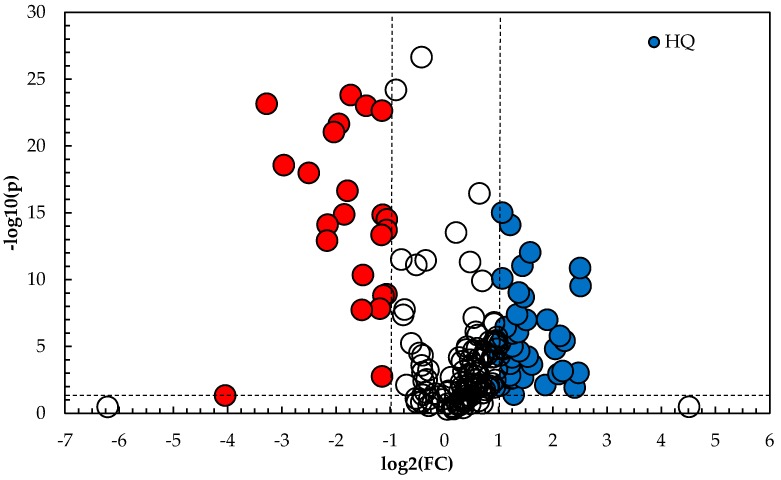
Volcano plot of the analyzed lavender oil samples by untargeted GC-MS.

**Figure 5:**
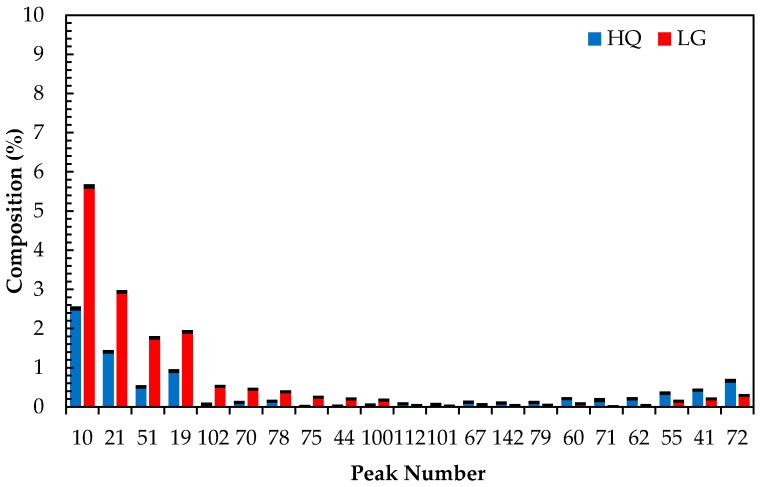
Top 15 significant peak features identified in the volcano plot of the analyzed lavender oil samples by untargeted GC-MS.

The objective of the PLS-DA analysis was to increase the predictive capability of the model, in addition to identify the peaks that provide the greatest differentiation between the high quality and lower grade oil samples. As such, the R^2^*X*, R^2^*Y* and *Q*^2^ values for the PLS-DA model were 0.903, 0.990 and 0.970, respectively, thereby providing greater predictability than the model created using targeted data. Plotting a volcano plot of the −log_10_ *p*-value for each peak detected against the log_2_ Fold Change (FC) displays the GC-MS data in way that clearly distinguishes the two cohorts and identifies the features that explain the differentiation between the sample cohorts. Figure 4 presents the volcano plot of the GC-MS data, and Figure 5 presents the top 15 compounds identified as a result of the volcano plot. It is noted that these compounds could not be identified using the Adam’s essential oils reference library with any real confidence (library match ≥70%) and additional work is needed to identify and characterize them in terms of lavender quality. However, for the work presented in this paper, their identification is not necessary as it will have no bearing on the effectiveness of the developed PLS-DA model to predict which cohort an unknown sample belongs to.

### 2.4. Characterisation of Unknown Samples

In order to evaluate and assess the predicative capability of the developed PLS-DA model, 9 unknown samples were analyzed according to the method described herein and applied to the PLS-DA model. As illustrated in Table 3, the three unknown samples either strongly correlated with the high quality or lower grade oil cohorts. Correlation for the high quality oils are observed when the YPredPS (Y Predicted list for PLS-DA model) value is nearest to 1, where 1 is defined as being equal to the cohort reference material. Considering the oils sampled are natural, some variation is to be expected. As evident in Table 3, six of the unknown samples were characterized as belonging to the high quality oil cohort while three unknowns were characterized as belonging to the lower grade oil cohort. Furthermore, a misclassification analysis of the entire dataset indicates that 100% of the data was successfully classified, with a Fisher probability test of 7.1 × 10^−16^. It is important to note, the misclassification analysis includes the 30 high quality and 24 lower grade oil samples, in addition to the unknown samples. A summary of the misclassification analysis is presented in Table 4.

## 3. Materials and Methods

### 3.1. Sample Preparation

As the aim of this study was to rapidly assess the lavender oils received by essential oil distributors for the purpose to characterize and grade oils of varying quality, a distributor provided 54 lavender oil samples for characterization. Of these, 30 originated from France/Bulgaria and 24 originated from Indonesia/Cambodia. Nine additional oils of unknown origin (unclassified samples) were also provided for analysis.

Each sample was prepared by diluting 20 µL into 2.0 mL hexane (MS Grade, Sigma Aldrich, Castle Hill, NSW, Australia). A 50 ppm C_8_-C_22_ mixed alkane standard (Fluka, Castle Hill, NSW, Australia) was used as an internal standard. Each sample was analyzed in in triplicate and reported as the average response. 

### 3.2. Gas Chromatography-Mass Spectroscopy (GC-MS) Analysis

An Agilent 5973 GC-MS chromatograph fitted with a DB-5MS column (Agilent Technologies, Mulgrave, Australia), with an ID of 250 μm and film thickness (*df*) 0.25 μm. All injections were performed in split mode (40:1), with 0.2 µL volume; the injector was held at 220 °C. The oven initial temperature of 60 °C was increased to 246 °C at 3 °C/min. The transfer line was maintained at 280 °C and the source at 230 °C. Mass spectra were acquired from 40 to 415 *m/z* range, at an acquisition frequency of 4 spectra/s. Data acquisition and processing were performed by MassHunter (Agilent Technologies, Mulgrave, VIC, Australia). Pre-processing and peak alignment was performed in Mass Hunter (Version B.07.01/Build 7.1.524.0, Agilent Technologies, Mulgrave, VIC, Australia). Characterization of individual compounds in the reference oil was performed in triplicate by GC-MS with compound identification undertaken with Adams Essential Oil Quadrupole Mass Spectroscopy Library [25]. Statistical analysis was performed by SIMCA 14 (Umertics, Umea, Sweden). Peak features were considered significant when comprising a signal-to-noise (S/N) ratio greater than 50 (and *p*-values < 0.05).

## 4. Conclusions

The current standard test methods such as the ISO Standard 11024 for the identification of lavender oil cannot be expanded to reliably determine the quality classes of lavender oil. The reason for this is the application of limits to the percentage abundance of only seven (out of 170) identified compounds. This proved to be a drastic over simplification to differentiate between subtle differences in a very complex sample type. A better approach would be to expand the analysis to include all 170 identified compounds within the lavender oils and analyze the correlations between their percentage abundance.

The chemometric model developed utilized the 170 compounds and identified 15 unique compounds that greatly differentiate between the two classes of lavender oil but also displayed little inter-variation between samples of the same cohort. This enabled the rapid characterization of the oils between these two varieties to be undertaken using a PLS-DA predictive model without the issues of sample-to-sample variation that comes with biological samples, and as evident in the target analysis.

When an additional 9 unknown/unclassified lavender samples were subsequently analyzed and the chromatograms run against the predictive chemometric model, all 9 uncharacterized oils were correctly identified. A misclassification analysis was performed against the samples as well as the 54 samples used to train the model and it was found that the predictions were sound. This study has successfully created a predictive test for the rapid quality assessment of lavender essential oil by using a combination of GC-MS profiling and a chemometrics predictive modelling.

## Figures and Tables

**Table 1:** GC-MS characteristics of reference lavender oil sample.

Compound Name	LRI	RT (min)	Composition^ (%)	%RSD	ISO Lavender Oil Specification (%) *
1,8-Cineole	1042.86	12.83	1.46	3.04	NR
*cis*-β-Ocimene	1048.57	12.96	3.33	4.52	3−9
Linalool	1104.00	14.21	31.17	1.08	25−38
1-Octen-3-yl acetate	1105.00	14.24	1.64	1.51	<1.8
Camphor	1165.00	15.54	0.53	1.64	NR
Linalyl acetate	1253.68	17.40	36.55	1.62	25−45
Lavandulyl acetate	1283.16	18.01	1.86	1.34	>1.0

Note: RT is defined as retention time. ^Reference sample was analyzed in triplicate. * ISO standard 11024 [22,23]. NR is defined as “not reported”.

**Table 2:** Statistical figures of merit for the analyzed high quality and lower grade lavender oils.

	Statistical Figures of Merit													
	Linalyl Acetate		Linalol		Lavandulyl Acetate		1,8-Cineole		1-Octen-3-yl Acetate		Camphor		*cis*-β-Ocimene	
Statistics	HQ	LG	HQ	LG	HQ	LG	HQ	LG	HQ	LG	HQ	LG	HQ	LG
**Mean (mg/mL)**	**9.09**	**7.66**	**6.67**	**9.01**	**0.76**	**0.99**	**0.31**	**0.47**	**0.20**	**0.12**	**0.08**	**0.02**	**0.31**	**0.15**
**Std. Deviation (mg/mL)**	0.37	0.29	0.42	0.37	0.19	0.16	0.13	0.09	0.03	0.02	0.04	0.03	0.21	0.05
**%RSD**	4	4	6	4	24	16	41	20	14	18	45	13	69	34
**Std. Error (mg/mL)**	0.07	0.06	0.08	0.08	0.04	0.03	0.02	0.02	<0.01	<0.01	<0.01	<0.01	0.04	<0.01
***F*-Test Two-Sample for Variances**														
*F*	1.60		1.25		1.44		1.95		1.63		1.90		18.56	
*F*-crit.	2.07		2.07		2.07		2.07		2.07		2.07		2.07	
Ratio *	**0.77**		**0.60**		**0.69**		**0.94**		**0.79**		**0.92**		8.95	
***t*-Test: Two-Sample Assuming Unequal/Equal Variances**														
*t* stat	14.45		19.94		4.13		4.83		11.13		11.47		3.84	
*t*-crit.	2.01		2.01		2.01		2.01		2.01		2.01		2.04	
Ratio^^^	**7.18**		**9.91**		**2.05**		**2.40**		**5.53**		**5.70**		**1.88**	
**Anova: Single Factor**														
*F*	208.72		397.55		17.03		23.32		123.97		131.50		11.69	
*F*-crit.	4.05		4.05		4.05		4.05		4.05		4.05		4.05	
Ratio^#^	**51.51**		**98.12**		**4.20**		**5.76**		**30.60**		**32.45**		**2.89**	

Note: HQ is defined as an oil characterized as “High quality”; LG is defined as an oil characterized as “Lower grade”. * If the *F*-Test Two Sample for Variances ratio of *F*:*F* crit. one-tail is greater than 1, then the sample populations are unequal. ^ If the *t*-Test: Two-Sample Assuming Unequal/Equal Variances ratio of t-Stat:t crit. two-tail is greater than 1, then the observed difference in sample mean is significant. # If the ANOVA: Sing Factor ratio of *F*:*F*-crit. is greater than 1, then the sample cohorts are considered different. The α (significance level) used to obtain all the critical values was 0.05.

**Table 3:** Predictive component of the PLS-DA model when applied for three unknown lavender oil samples.

Sample Type	ID	PLS-DA Predictive Component				Prediction
		YVarPS (HQ)	YPredPS (HQ)	YVarPS (LG)	TPredPS (LG)	
Reference	A	1	1.031	0	−0.031	
	B	1	1.002	0	−0.002	
	C	1	0.996	0	0.004	
Unknown	1	--	0.021	--	0.979	LG
	2	--	0.991	--	0.009	HQ
	3	--	0.998	--	0.002	HQ
	4	--	0.001	--	1.001	LG
	5	--	1.017	--	−0.017	HQ
	6	--	0.927	--	0.073	HQ
	7	--	0.997	--	0.003	HQ
	8	--	−0.055	--	1.055	LG
	9	--	0.948	--	0.052	HQ

Note: HQ is defined as an oil characterized as “High quality”; LG is defined as an oil characterized as “Lower grade”.

**Table 4:** Misclassification analysis of the analyzed lavender oil samples by GC-MS.

Oil Type	Members	Correct	In Essence	Oil Garden	No Class
In Essence	30	100%	30	0	0
Oil Garden	24	100%	0	24	0
No class	9	-	6	3	0
Total	63	100%	36	27	0
